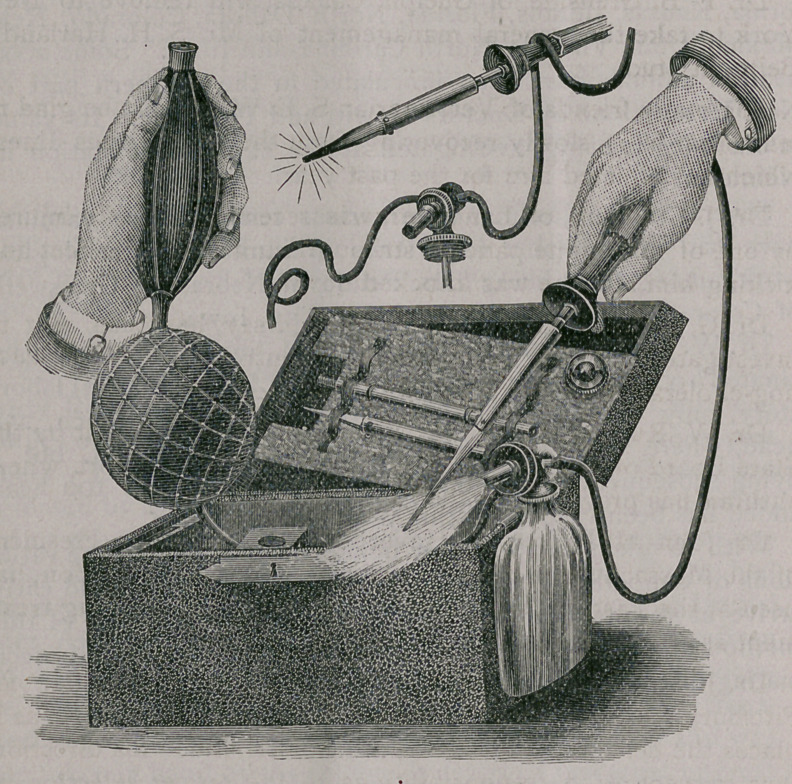# Editorials

**Published:** 1895-09

**Authors:** 


### EDITORIAL.

### ASSOCIATION MEETINGS.

SEPTEMBER of this year will prove a phenomenal month for important veterinary association meetings, and we devote in this number a goodly portion of our pages to a brief prospectus of these very important gatherings. Many of them are to be held just prior to the annual gathering of the U. S. V. M. A. at Des Moines. Commencing with Pennsylvania, then Missouri, New York, Iowa, and California, with several local associations, which promise that the winter's work shall be well begun.

These various State meetings will all send delegates to Des Moines, and thus add strength and value to the national meeting. Many of the leading questions of the day will be discussed in home circles first and find their fruition at the parent meeting. Every veterinarian should at least attend his home gathering, and there aid in better equipping their representatives at Des Moines. The weight of influence represented by these various gatherings will prove a potent factor in strengthening every progressive movement in the many directions of work to which the national association has given impetus during the past ten years.

The question for each veterinarian to decide in regard to these meetings is not, Can I afford to go? but, Can I afford to stay away? Can I afford to lose the valuable papers and discussions that will come up? This is specially true in regard to the U. S. V. M. A., where for the first time in many years the major portion of the programme will be taken up by subjects of every-day practical importance to the routine practitioner.

### UNIFORM VETERINARY DEGREE.

WE are glad to be privileged this month to publish a very important communication on the subject of a uniform degree or title for the veterinarian from so able, impartial, and yet interested writer as Professor C. W. Stiles. It comes to us at an opportune moment, on the eve of the consideration of this subject at the meeting of the Association of Veterinary Faculties at Des Moines, on the evenings of September 10th and 11th.

### EDITORIALS IN "AMERICAN VETERINARY REVIEW," AUGUST, 1895.

It is not often that we honor with notice any articles of a personal nature or that we consider it within our dignity to enter into any personal controversy, but certain editorials in the August number of the *American Veterinary Review* (which undoubtedly reaches a certain number our readers) are so misleading in their statements that we feel called upon to correct them.

Under the article, "Veterinary Educational Progress(?) in New York State," the *Review* states that the Board of Regents "very properly applied to the official representative of the profession in the Empire State, and so the New York State Veterinary Medical *Association* was *asked* to nominate ten members," etc. If the *Review* will read the plain English of the law it will learn that the law *requires* the nominations to come from the New York State Veterinary Medical *Society*. There were, therefore, no option on the part of the Board of Regents. Considering that Dr. Hinkley is the only editor whose name appears on the cover of the *American Veterinary Review* who-ever attended more than one of the meetings of the State Society, it is not to be wondered at that the *Review* did not know the proper title of the *Society*.

The *Review* states that "one of the plainest propositions to those who had contemplated the new law was that certainly no member of the faculty of any school," etc., etc. Will the *Review* inform us, who connected with it (Dr. Hinkley excepted), or with the American Veterinary College, ever spent one minute or one dollar in aid of the recent legislation in New York State? On the contrary, can the *Review* deny that one of its editors, not an American citizen, did not use endeavors to interfere with the recent legislation at Albany?

If but "a handful," as the *Review* terms them, came to the special meeting of the New York State Society, that handful represented the men through the State who for years have been spending time and money in the advancement of the Society.

The *Review* states that, after a recess, the meeting rescinded a resolution of the morning session before "*certain opponents*" had returned to the hall. The *certain opponent* who interfered with the morning session of the special meeting and did not return to the afternoon session was, as the *Review* may *not* know, a person who had not paid his dues to the Society for several years and who was not eligible either to speak or vote at the meeting. Any "mischief done legally" was the carelessness of the President and Secretary in not remembering that said *certain opponent* had no rights at the meeting, and in their courtesy to him as an old man.

The *American Veterinary Review* may be accustomed to associate with a class of men who, because they are interested in one lot of students, cannot be honest and just in their examination of others. We, in our acquaintance among teachers in many colleges, and in our friendship in the New York State Veterinary Medical Society, fortunately know a class of men whom we know to be honest under any circumstances and in whom we can repose a trust, as has been done by the New York State Society.

In another editorial, a sequel of one which appeared a month or two ago, the *Review* addresses its subscribers and pleads charity from them on account of the difficulties and financial trouble of a firm who published the *Review* and were part owners. Does the *Review* forget its origin? The *Review* was originated by the United States Veterinary Medical Association. During the first years of the *Review* its deficit in expenses was paid by the United States Association. When the *Review* had been established on a paying basis, at the expense of the United States Association, it was presented to Dr. Liautard free from debt, and with the friendship of every member of the Association. When it became the organ of a college and was transferred to the hands of the now defunct publishing firm and was again taken in hand by the college, it deserved just what interest and sympathy it merits on its present deeds, but none whatever as a business enterprise.

The editors of the JOURNAL have spent far more money for the benefit of the profession than the *Review* has ever done. The JOURNAL undoubtedly was careless in its publication under the last management, but not more so than were its subscribers in the payment of their bills. But to-day under the present liberal management, independent, as the name of Dr. Hoskins guarantees, from any influence but that of the general good of the profession at large, the JOURNAL can promise more liberal veterinary information than any other publication.

### ANIMAL VACCINATION.

THE value of the various vaccine viruses on American soil will soon be measured from a practical point of view. The Pasteur Anthrax Vaccine Company, of New York, representing the parent company of Paris, France, have now a force of veterinarians working in the States of New Jersey, Texas, California, Kansas, Illinois, Florida, North Dakota, Colorado, New Mexico, Utah, Virginia, North Carolina, and Iowa. Some 2000 head of animals have been inoculated in the southern part of New Jersey alone by Doctors Hewitt and Tremaine. Its value in black-leg is being investigated at the experiment station in Nebraska.

Several of the leading veterinarians testing these vaccines have had considerable experience in sanitary bacteriological work, among them being Drs. Peters, of Nebraska; Gresswell, of Colorado; and Johnson, of Sioux City, Iowa.

Dr. W. J. Oliver, of Los Angeles, Cal., in March, 1894, vaccinated 190 cattle with vaccine from the Paris (France) laboratory, only one animal dying. At the Cerristor Ranch, 258 head, where over 40 had died prior to vaccination, but 3 dying subsequent to the test. The same herd with 100 head added has been vaccinated this year with no losses.

With so much territory now being covered, with all the variations of temperature, situation, and conditions, we should early arrive at results that will decide the permanent value of this method of dealing with these peculiarly fatal and rapidly decimating diseases of our flocks and herds.

---

### TO OUR CONTRIBUTORS AND READERS.

WE regret exceedingly that we are forced to postpone the publication this month of an unusual amount of valuable and interesting material, though we are giving to our readers twelve pages extra of reading matter. Among the matter laid over are a number of original articles and many important selections, abstracts, and communications. We wish at this time to express to our contributors our warmest appreciation of their great kindness and assistance in aiding the new management of the JOURNAL, and thus contributing so much to the strengthening of American veterinary literature.

### AN IMPROVED VETERINARY THERMO-CAUTERY.

WE are pleased to be able this month, through the kindness of Messrs. Teuful & Brother, to display an improved thermo-cautery that covers many of the objections and annoyances of those now in use. It does away with the alcohol lamp, where it formerly was necessary to heat the point and then in transferring the rubber tubes to the benzine reservoir lose the heat quite frequently. A combination benzine reservoir with blast-lamp attachment obviates the need of alcohol lamp, thus facilitating the rapid heating of cautery points and simultaneous injection of hydrocarbon gases into the heated platinum points. This is done by a simple and ingenious contrivance screwed on to the metallic benzine bottle. By regulating this valve attachment the platinum points may be kept at white or red heat as desired. The platinum points of firing-irons are made removable by unscrewing.

## Figures and Tables

**Figure f1:**